# Supplementary figures and images for: Association between Tumor Mutational Burden, Stromal CD8+ Tumor-Infiltrating Lymphocytes, and Clinical Factors in Cervical Cancers Treated with Radiotherapy
Source: Cancers (Basel). 2023 Feb 14;15(4):1210. doi: 10.3390/cancers15041210 (PMC9954714; doi:10.3390/cancers15041210)

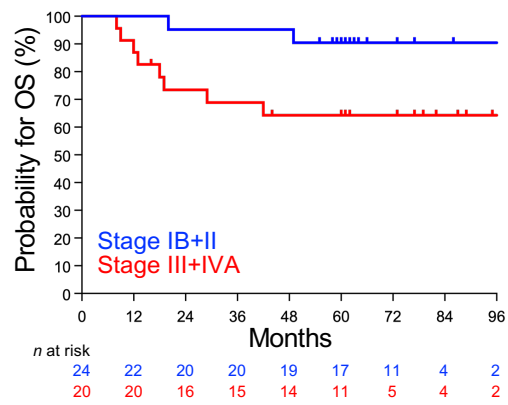

**Supplementary Figure S1. Kaplan–Meier estimates for OS, stratified by FIGO staging.**

Supplement: Supplementary file 1 [file cancers-15-01210-s001.zip › cancers-2176834-supplementary.pdf]
